# Young at Heart: Combining Strategies to Rejuvenate Endogenous Mechanisms of Cardiac Repair

**DOI:** 10.3389/fbioe.2020.00447

**Published:** 2020-05-13

**Authors:** Edoardo Maghin, Patrizia Garbati, Rodolfo Quarto, Martina Piccoli, Sveva Bollini

**Affiliations:** ^1^Tissue Engineering Laboratory, Fondazione Istituto di Ricerca Pediatrica Città della Speranza, Padua, Italy; ^2^Department of Women’s and Children Health, University of Padova, Padua, Italy; ^3^Regenerative Medicine Laboratory, Department of Experimental Medicine, University of Genova, Genova, Italy; ^4^UOC Cellular Oncology, IRCCS Ospedale Policlinico San Martino, Genova, Italy

**Keywords:** paracrine, extracellular matrix, decellularization, cardiac repair, regeneration, extracellular vesicles, stem cell

## Abstract

True cardiac regeneration of the injured heart has been broadly described in lower vertebrates by active replacement of lost cardiomyocytes to functionally and structurally restore the myocardial tissue. On the contrary, following severe injury (i.e., myocardial infarction) the adult mammalian heart is endowed with an impaired reparative response by means of meager wound healing program and detrimental remodeling, which can lead over time to cardiomyopathy and heart failure. Lately, a growing body of basic, translational and clinical studies have supported the therapeutic use of stem cells to provide myocardial regeneration, with the working hypothesis that stem cells delivered to the cardiac tissue could result into new cardiovascular cells to replenish the lost ones. Nevertheless, multiple independent evidences have demonstrated that injected stem cells are more likely to modulate the cardiac tissue via beneficial paracrine effects, which can enhance cardiac repair and reinstate the embryonic program and cell cycle activity of endogenous cardiac stromal cells and resident cardiomyocytes. Therefore, increasing interest has been addressed to the therapeutic profiling of the stem cell-derived *secretome* (namely the total of cell-secreted soluble factors), with specific attention to cell-released extracellular vesicles, including exosomes, carrying cardioprotective and regenerative RNA molecules. In addition, the use of cardiac decellularized extracellular matrix has been recently suggested as promising biomaterial to develop novel therapeutic strategies for myocardial repair, as either source of molecular cues for regeneration, biological scaffold for cardiac tissue engineering or biomaterial platform for the functional release of factors. In this review, we will specifically address the translational relevance of these two approaches with *ad hoc* interest in their feasibility to rejuvenate endogenous mechanisms of cardiac repair up to functional regeneration.

## *Divide and Conquer* Cardiac Repair and True Heart Regeneration

Cardiovascular disorders significantly affect life expectancy; according to the World Health Organization (WHO), by 2030 about 23 million people annually will be severely affected by heart failure (Leone et al., 2015; Mozaffarian et al., 2015; Benjamin et al., 2018). Cardiac dysfunction may arise by significant loss of resident cardiomyocytes. Indeed, prolonged interruption of coronary blood circulation can cause myocardial infarction (MI) with consequent cardiomyocyte irreversible damage, leading to the development of fibrotic tissue that replaces the contractile myocardium. The injured heart can activate limited wound healing as life-saving mechanism to avoid cardiac rupture. This leads to maladaptive ventricle remodeling and compensatory cardiac hypertrophy leading to heart failure over time (Frantz et al., 2009). Despite significant improvements, interventional cardiology and prompt pharmacological treatments after MI cannot reverse cardiac damage, as they may only minimize cardiomyocyte death or delay heart failure onset (Ezekowitz et al., 2009). Thus, the most effective therapeutic approach is still represented by heart transplantation, with severe limitations due to donor organ availability and compatibility (Hsich, 2016). Since there is an unmet clinical need for current therapies to replenish loss of cardiomyocytes and vasculature, many efforts have been currently focusing on defining novel therapeutic strategies to implement myocardial repair and regeneration.

Notably, full regeneration of the injured heart is a well-established process in lower vertebrates, as opposite to the adult mammalian myocardium. The teleost fish has shown of functional cardiac reconstitution following injury, by means of replicating cardiomyocytes replacing a temporary scar within 1–3 months (Kikuchi et al., 2010; Han et al., 2019). Nevertheless, it has been recently reported that in adult humans *de novo* generation of cardiomyocytes can actually occur, although via very low self-renewal rate (ca. 0.5–1% per year), which is not therapeutically relevant *per se* (Bergmann et al., 2009; Senyo et al., 2013). Recent evidences that the neonatal rodent heart can still harbor significant cardiomyogenic potential has further driven the attention toward cardiomyocyte proliferation as *bona fide* regenerative mechanism. Within the first week of life, neonatal mice can almost entirely regenerate their heart after severe injury via renewal of surviving cardiomyocytes (Porrello et al., 2011, 2013; Mahmoud et al., 2014). After this short post-natal window, there is a clear transition from regeneration into scarring and fibrosis, as the typical molecular signature of the mature adult cardiac wound healing response (Aurora et al., 2014). Likewise, functional recovery of the myocardial tissue in a unique case of a human newborn undergoing severe MI by coronary artery occlusion has been recently reported; similarly to the neonatal rodent heart, the child showed rescue of cardiac function within weeks via putative cardiac regeneration (Haubner et al., 2016).

Evidences that the mammalian heart possesses some restorative potential have been supported by the identification of resident stromal mesenchymal cells, defined as cardiac progenitor cells (CPC). CPC were originally reported in 2003 when describing Lin^–^c-kit^+^ cardiac stem cells acquiring phenotypic features of cardiomyocyte-like cells (Beltrami et al., 2003). Since then, different populations of endogenous CPC have been described, such as c-kit^+^, Sca-1^+^, cardiosphere-derived CPC and epicardium-derived progenitor cells (EPDC). All such stem-like cell populations can be isolated from discarded tissue obtained during heart surgery or endocardial biopsy according to different protocols, as extensively reviewed in Bollini et al. (2011b). While some CPC, like EPDC, may harbor some degree of vascular and cardiomyocyte plasticity during embryonic development (Zhou et al., 2008), generally speaking they become quiescent soon after birth, unless stimulated by injury (Lepilina et al., 2006; Limana et al., 2010; Huang et al., 2012). Initially, great excitement was addressed toward these cells as stem-like progenitors with cardiac-specific differentiation potential within the adult heart. Yet, multiple independent lines of investigation have recently questioned the cardiomyogenic and/or cardiovascular commitment of several adult CPC subpopulations (i.e., c-kit- and Sca-1-positive ones, Sultana et al., 2015; Kanisicak et al., 2017; Li et al., 2018; Tang et al., 2018; Zhang L. et al., 2018) with serious expression of concerns and retractions of several studies (Estes Mark, 2019). Despite the ongoing controversy on CPC cardiomyogenic conversion, CADUCEUS and ALLSTAR clinical trials based on autologous and allogeneic cardiosphere-derived cell therapy proved to be safe and resulted into some degree of improved viable heart mass and contractility in patients with MI and left ventricle dysfunction (Makkar et al., 2012; Chakravarty et al., 2017). Therefore, it seems reasonable that adult CPC may still contribute to improve heart function – although unlikely via direct differentiation- and represent a therapeutic target to optimize cardiac repair. Additionally, activation of resident CPC following injury showed to be much stronger and responsive in the neonatal mouse heart compared to the adult (Jesty et al., 2012).

Being loss of myocardial tissue the main limiting factor for heart function, preclinical research has been lately focused on two biological strategies to improve endogenous mechanisms of cardiac repair and regeneration: (*i)* preservation of viable myocardium during injury or disease and (*ii)* replacement of cardiomyocytes to restore structural and functional integrity of the damaged heart (Broughton et al., 2018). The first one is based on enhancing cardio-protection so to counteract pathological remodeling; this can be mainly achieved via prompt *in situ* inhibition of cardiomyocyte apoptosis and/or their premature senescence, by quenching prolonged inflammation, while supporting *de novo* local angiogenesis and (re)activating endogenous CPC (Prabhu and Frangogiannis, 2016; Spath et al., 2016; Huang and Frangogiannis, 2018; Li et al., 2019). The latter, which specifically targets myocardial renewal, truly represents the *sine qua non condition* to conquer true cardiac regeneration via restoration of active cardiomyocyte cell division (Cahill et al., 2017; Eschenhagen et al., 2017; Wang et al., 2017; Vujic et al., 2020). Indeed, increasing efforts have been lately dedicated to define putative therapeutic strategies to resurge myocardial renewal in the adult heart. Yet, it remains quite controversial to provide satisfactory and reliable validation of true cardiomyocyte division by means of cytokinesis, over more limiting binucleation or polyploidy, as extensively reviewed in Leone et al. (2015) and Leone and Engel (2019). Moreover, microRNA therapy via viral vector has recently shown to successfully target cardiomyocyte de-differentiation and proliferation in a big preclinical animal model of MI, resulting in remarkable myocardial regeneration; nonetheless, *de novo* proliferating cardioblasts expressed an immature phenotype and caused lethal arrhythmia, indicating that their genetically induced constitutive stimulation may not be indicated, if timely control of such renewal mechanism cannot be provided (Gabisonia et al., 2019).

**FIGURE 1:**
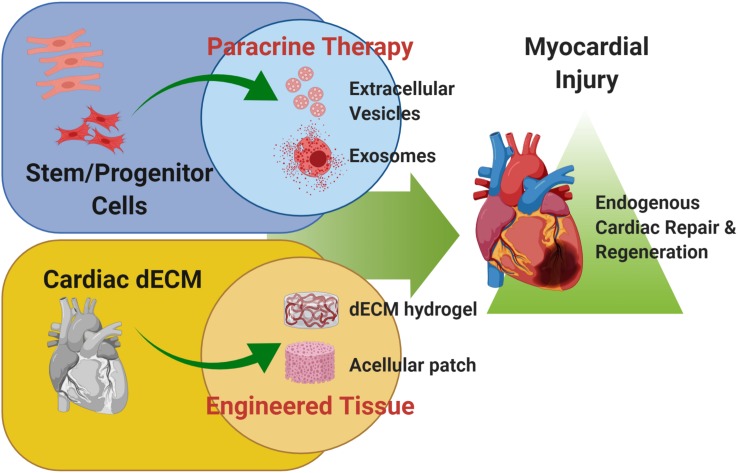
Cardiac regenerative strategies. Schematic representation of the main experimental cardiac medicine approaches suggested to address myocardial injury and aiming at stimulating endogenous mechanisms of repair and myocardial restoration by means of stem cell-derived paracrine effectors and biomaterials. Schematic was made using BioRender (https://app.biorender.com).

These evidences suggest that the mammalian heart can harbor some intrinsic regenerative capacity, based on reparative and renewal mechanisms; these are broadly active during embryonic development up to early neonatal stages. Such restorative program is transient, being lost after the first week of birth, suggesting a sort of “memory loss.” Therefore, preclinical cardiovascular research has lately focused on alternative strategies to “rejuvenate” the forgotten endogenous potential of adult heart, including (a) the stimulation with stem/progenitor cell-derivatives and (b) the innovative use of cardiac decellularized extracellular matrix (dECM, Figure 1). In this review, we will provide an overview of these two possible experimental approaches to resurge intrinsic mechanisms of cardiac repair and myocardial renewal.

## Say It Right: at the Heart of Inter-Cellular Paracrine Communication

Data from clinical and preclinical studies on stem cell-based therapy for cardiac disease have shown that cells transplanted into compromised myocardium are very unlikely to contribute to novel cardiovascular cells by means of differentiation (Murry et al., 2004; Noiseux et al., 2006; Langrzyk et al., 2018). Multiple independent analyses have also revealed that transplanted cells fail to engraft and survive in the long term (Freyman et al., 2006; Schachinger et al., 2008; Noort et al., 2010). Nonetheless, in most preclinical studies, they concurred to counteract worsening of cardiac function via beneficial paracrine effects acting on the local cardiac microenvironment, such as supporting resident cardiomyocyte survival and local angiogenesis, while modulating acute inflammation and limiting fibrosis (Gnecchi et al., 2006; Mirotsou et al., 2007, 2011). Indeed, there is common consent that different stem and progenitor cells secrete an extensive variety of trophic factors, as chemokines, cytokines, growth factors and extracellular matrix (ECM) molecules, which can influence and change the environment composition, thus modifying the neighboring cells behavior (Korf-Klingebiel et al., 2008; Baraniak and McDevitt, 2010; Hodgkinson et al., 2010; Albulescu et al., 2015). Such paracrine capacity may represent an appealing and useful tool for the development of innovative regenerative medicine strategies. As consequence, paracrine modulation of cardiac tissue by stem cell-secreted molecules has recently appeared as a promising tactic for boosting cardiac healing with rising attention toward the functional characterization of the stem/progenitor cell “secretome,” as the growth factors, chemo-attractant molecules and extracellular vesicles (EVs) released by paracrine secretion (Hodgkinson et al., 2010). In this perspective, several studies have been reported proof-of-principle cardiac paracrine therapy, via the administration of different cell-conditioned media recapitulating the cardio-active beneficial effects of the secreting cells, including also the reinstatement of endogenous stromal cardiac progenitor embryonic potential and the restoration of cell cycle activity in resident cardiomyocytes (Hatzistergos et al., 2010; Nguyen et al., 2010; Bollini et al., 2011a; Timmers et al., 2011; Hynes et al., 2013; Yang et al., 2013; Danieli et al., 2015; Rao et al., 2015; Hodgkinson et al., 2016; Lazzarini et al., 2016; Balbi et al., 2019).

From a translational point of view, the stem/progenitor cell secretome may represent an appealing ready-to-use *advanced therapy medicinal product* (ATMP) that could be manufactured via scale-up *in vitro* culture systems. Therefore it could offer the big advantage of being promptly accessible to cardiovascular patients, whenever needed; moreover, this strategy may overcome limits and concerns related to canonical cell therapy, including, for instance, donor cell engraftment, immune-compatibility and cost-effective, time-consuming procedures to provide an high amount of cells to be injected (Segers and Lee, 2008; Malliaras and Marban, 2011). Since paracrine therapy may represent a working tactic to resurge the neglected intrinsic potential of adult heart, the hunt is now on finding the most feasible stem cell source to be exploited for cardiac repair and regeneration. In such perspective, ease of isolation and proliferative potential, along with cardio-active secretory profile are strategic features for the ideal stem/progenitor cell candidate for paracrine therapy.

Different somatic stem cells have been investigated with studies mostly referring to mesenchymal stromal cells (MSC) as suitable source. MSC with relevant paracrine potential for cardiovascular disease have been described as isolated from either bone marrow or adipose tissue, which represent easily accessible cell options. Likewise, fetal and perinatal MSC obtained from extra-embryonic annexes such as placenta tissue (Chen et al., 2015; Danieli et al., 2015; Van Linthout et al., 2017; Bier et al., 2018), umbilical cord (Corrao et al., 2013; Jin et al., 2013; Lim et al., 2018; Zhao et al., 2019) and amniotic fluid (Bollini et al., 2011a; Lazzarini et al., 2016; Balbi et al., 2019) have been reported as endowed with interesting paracrine potential in the cardiovascular field, as extensively reviewed in Bollini et al. (2018a). More recently, endogenous CPC have also been broadly investigated as exploitable options. While general consensus on adult CPC debatable differentiation capacity has not been reached, multiple studies confirmed their modulatory beneficial effects; these seems to be mediated by secreted soluble factors improving cardiac repair, as main functional mechanism of action (Barile et al., 2017a; Sharma et al., 2017; Broughton et al., 2018; Micheu et al., 2018; Pagano et al., 2018; Rafatian and Davis, 2018; Mardanpour et al., 2019). All such cell populations present different advantages and/or limitations, due to their properties. For instance, human adult somatic sources may be affected by low yield, invasive sampling, *in vitro* controversial self-renewal and phenotypic drifting due to donor age. On the other hand, more developmentally immature stromal cells offer a suitable alternative. These include progenitors that can be obtained from fetal and perinatal leftover samples of prenatal diagnosis (i.e., villi and amniotic fluid) or clinical waste material at birth (placenta membranes, amniotic fluid and umbilical cord tissue); specifically, they are endowed with remarkable proliferative potential along with ease of isolation, while avoiding ethical issues, as extensively reviewed in Bollini et al. (2018a, b). Interestingly, CPC have been broadly described to act as paracrine master regulators of cardiomyocyte proliferation during embryonic cardiac development by releasing mitogens targeting the underlying developing myocardium (Smith and Bader, 2007; Lavine and Ornitz, 2008). As well, they showed to underpin restorative responses within the injured heart of regenerative preclinical models (i.e., zebrafish and the mammalian neonatal heart), by locally instructing neighboring cardiovascular cells (Lepilina et al., 2006; Jesty et al., 2012; Simpson et al., 2012; Hesse et al., 2014; Wehman et al., 2017). Yet, despite such appealing paracrine profile, mammalian adult CPC become almost completely unresponsive after birth and need substantial stimulation to be reactivated following injury, thus making their isolation and further *in vitro* amplification challenging (Smart et al., 2011; Dube et al., 2017; Balbi et al., 2019).

### Stem and Progenitor Cell-Extracellular Vesicles as Functional Messengers

Within the stem cell paracrine scenario, increasing attention has turned toward cell-secreted EVs, as functional biological conveyors of modulatory influence. EVs are phospholipid micro- and nano-vesicles that act as key mediators of inter-cellular communication affecting cellular functions. EVs are very heterogeneous and can be further sub-classified based on their size: from nano-scaled exosomes (ranging from 35 to 150-200nm) to medium-sized micro-vesicles (from 200 to 500nm) up to apoptotic bodies (>500 nm) (Thery et al., 2018). EVs carry a molecular cargo enriched with different bioactive factors (i.e., proteins, biolipids), as well as genetic information (more commonly non-coding RNA, such as microRNA, miRNA) (Teplitz, 1990).

EVs are secreted by different sources including cardiac, endothelial and inflammatory cells, advocating their relevant function in the cardiovascular system, especially within the damaged heart (Barile et al., 2014; Sluijter et al., 2018). Stem- and mesenchymal stromal cell-derived EVs have been shown to influence the immune system by modulating natural killer (NK) cells, dendritic cells (DC), monocytes/macrophages, microglia, T and B cells (Xie et al., 2020); indeed, MSC-derived EVs containing anti-inflammatory interleukin-10 (IL-10) and transforming growth factor beta 1 (TGF-β1) reduced the NK release of interferon gamma (INF-γ) and tumor necrosis factor alpha (TNF-α) release, thus alleviating the inflammatory response in a graft-versus-host disease model (Kordelas et al., 2014). Stem cell-derived EVs have also demonstrated to influence the skewing of macrophages toward a pro-resolving phenotype (Hyvarinen et al., 2018), as well as by reducing their *in vivo* infiltration along with quenching of severe inflammation following skeletal muscle tissue damage (Balbi et al., 2017; Lo Sicco et al., 2017). Moreover, human CPC-EVs delivered locally to the injured murine myocardium, either during the acute or chronic inflammatory phase, resulted in significant reduction of pro-inflammatory macrophages, neutrophils and circulating cytokines (Cambier et al., 2017; Harane et al., 2020). Of note, stem and progenitor cell-derived EVs have been broadly described as positively influencing physiological pathways involved in cardioprotective and tissue regeneration mechanisms (Baraniak and McDevitt, 2010). Both MSC- and CPC-derived EVs exerted cardio-active beneficial effects in preclinical animal models of myocardial injury, by more sustained influence – over their secreting parental cells - in enhancing reparative mechanisms (Shao et al., 2017) (Figure 1). Indeed, EVs delivered to the injured heart demonstrated to trigger relevant pro-survival effects as preserving more viable myocardial tissue, while decreasing fibrosis via the activation of specific signaling pathways, including those regulated by Wnt/β-catenin and AKT (Barile et al., 2014; Cui et al., 2017). Moreover, small EVs isolated from human fetal amniotic fluid progenitor cells have recently showed to protect the cardiac tissue in a rat preclinical model of ischemia/reperfusion injury when administered systematically; although they did not show clear cardioprotective or angiogenic effects *in vitro*, they expressed significant chemotactic influence on endothelial cell migration via phosphatidylinositol 3-kinase (PI3K) signaling (Takov et al., 2020). CPC-EVs have been reported to reprogram dermal fibroblast to express antifibrotic, antiapoptotic, and pro-angiogenic potential and prime them toward a cardioprotective profile when transplanted into a preclinical rodent model of MI (Tseliou et al., 2015). Interestingly, proteomic profiling of human CPC-EVs indicated in the pregnancy-associated plasma protein-A (PAPP-A) a molecular candidate for their cardioprotective potential; PAPP-A was demonstrated to instruct vesicle release of insulin-like growth factor-1 (IGF-1) via cleavage of IGF-binding protein-4 (IGFBP-4), resulting in AKT and ERK1/2 phosphorylation in target cardiomyocytes, with marked pro-survival effect (Barile et al., 2018). The Notch pathway has also been indicated as potential mediator of MSC-EVs in exerting pro-angiogenic effects for the treatment of ischemia-related disease; EVs released by HIF-1α-overexpressing MSC were enriched with the Notch-ligand Jagged 1 and triggered angiogenetic responses on *in vitro* cultured endothelial cells (Gonzalez-King et al., 2017). Likewise, EVs secreted by Notch1-overexpressing cardiac stromal cells were highly cardioprotective and influenced resident cardiomyocyte cell cycle progression in a preclinical mouse MI model (Xuan et al., 2020).

A growing number of studies have also indicated a paracrine EV mechanism of action in the horizontal delivery of their RNA cargo to target cardiovascular cells. Y RNA fragment has been described as one of the most abundant RNA within cardiosphere-derived cells-EVs inducing a cardioprotective phenotype in target macrophage with secretion of anti-inflammatory IL-10 (Cambier et al., 2017). Similarly, several miRNAs have been associated with stem/progenitor-EV anti-apoptotic, proliferative and angiogenic effects, including – but not limited to - miR-210 and miR-146a (Barile et al., 2014, 2017b; Ibrahim et al., 2014; de Couto et al., 2017; Zhu et al., 2018; Milano et al., 2019). Stem cell-EVs, such as those isolated from immature amniotic fluid derived-MSC, also showed interesting “rejuvenating” effects, like *in situ* re-activation of endogenous CPC and the stimulation of resident cardiomyocytes progression within cell cycle stages after myocardial injury (Balbi et al., 2019). In particular, human amniotic fluid stem cell-EVs injected locally into the ischemic myocardium soon after MI, were able to trigger epicardium-derived progenitor cells to re-express the embryonic key gene *Wt1* as master regulator of their developmental juvenile potential (Smart et al., 2011; Bollini et al., 2014). The reactivated resident epicardial CPC did not show any sign of commitment toward either cardiomyogenic or cardiovascular lineages and disappeared after 4 weeks from treatment. Nonetheless, further *in vitro* investigation revealed that human epicardial CPC primed with human amniotic fluid stem cell-conditioned medium (containing EVs), produced a pro-angiogenic secretome driving tubulogenesis in HUVEC cells (Balbi et al., 2019). These results confirmed previous findings describing how severe myocardial injury can induce epicardial CPC proliferation, without differentiation into cardiomyocytes or endothelial cells, but with restoration of their paracrine activity on local *de novo* vascular network expansion (Zhou et al., 2011; Dube et al., 2017). In light of these evidences, endogenous CPC may represent an appealing therapeutic target for stem/progenitor cell-EVs for improving endogenous (paracrine) mechanisms of cardiac repair, as suggested by independent investigators (Alibhai et al., 2018).

Despite future paracrine therapy holds appealing potential for cardiovascular disease and heart failure, it may be challenged by some technical key aspects. Paracrine effects are limited in time *per se*, as swiftly impacting on the tissue micro-environment when released; hence, to significantly sustain reparative/regenerative mechanisms, multiple follow-up administration may be required. EVs isolated from human amniotic fluid-progenitors have shown to be effective in quenching skeletal muscle damage in a preclinical mouse model of muscle atrophy; yet, their beneficial effect was highest within 24 h from administration to then rapidly decrease within a week (Balbi et al., 2017). Interestingly, the same EVs, when delivered locally via intra-myocardial injection into a preclinical MI mouse model immediately after coronary ligation, were able to provide beneficial long-term effects in supporting cardiac function and counteracting pathological remodeling. This may suggest that, they may instruct resident cells to activate long-lasting responses, by promptly acting in the acute setting (Balbi et al., 2019).

Another relevant aspect to consider for the clinical translation of EV biology is the need for standard operative procedure for EV isolation to improve their yield and purity. Indeed, independent EVs preparations have shown different immunomodulatory potential (Kordelas et al., 2014). Likewise, reference guidelines for their *in vivo* administration and dosing are strictly required (Balbi et al., 2020; Yang et al., 2020). The International Society of Extracellular Vesicles (ISEV), has been addressing safety and regulatory requirements that must be considered for EV clinical application, as extensively reviewed in Thery et al. (2018). While an elegant study has recently described a CRISPR-Cas9-based readout system to investigate the regulatory mechanisms underlying EV-mediated RNA transfer between cells (de Jong et al., 2020), further investigation is still required to better elucidate EV targeting mechanism(s), along their pharmacokinetics and pharmacodynamics.

Considering these key aspects, it would be critical to take advantage of a controlled-release system to prolong the local administration of paracrine factors (such as EVs) within the injured myocardium. Systemic delivery of putative therapeutic paracrine factor(s), as the less invasive and more clinically compliant option, may be significantly restrained by the meager homing of the treatment to the cardiac tissue, as likely rapidly sequestered by off-target organs/tissues; thus, optimization of local administration would offer significant improvements.

## Decellularized Extracellular Matrix in Cardiac Repair and Regeneration

In order to implement heart regenerative strategies, devices made from ECM and other biomaterials were recently developed as systems capable of delivery therapeutic factors. These ECM-derived scaffolds, gels, and protein suspensions, are able to both convey cells and factors, and to enhance survival and regeneration of the heart. Importantly, significant therapeutic benefits were obtained when these ECM-derived scaffolds were used stand-alone in post ischemic models (Sarig et al., 2016). These studies have expanded the field of cardiac regenerative medicine, including preclinical therapeutic use of biomaterials and different ECM-derived formulations as reviewed in Spinali and Schmuck (2018), Tang-Quan et al. (2018), Xing et al. (2019).

Initially, tissue ECM was considered a biologically inert space, able to provide only a physical support to the attached cells. However, it is now clear that ECM is a dynamic and complex network of fibrous and adhesive proteins, serving as reservoir of different bioactive peptides and growth factors. Cell behavior is influenced by biochemical and biomechanical signaling present in the tissue microenvironment, including ECM, which is a dealer of cellular processes such as proliferation, differentiation, migration, and survival (Rao Pattabhi et al., 2014; Chen et al., 2018). In order to obtain ECM from a plethora of tissues and organs, decellularization protocols, by means of chemical/physical methods reaching the best compromise between the complete cell removal and the maintenance of the structural tissue proteins, are certainly the most used technique as reviewed in Gilbert et al. (2006), Song and Ott (2011), Keane et al. (2015), Taylor et al. (2017), Urciuolo and De Coppi (2018).

Within the cardiovascular scenario, a variety of naturally derived dECM sources have been investigated (with or without cells/factors) as cardiac patch or injected directly into the myocardium as hydrogel (Wainwright et al., 2010; Johnson et al., 2014). Since it was reported by the literature the importance of using tissue-specific derived biomaterials to obtain a precise and efficient organ regeneration, here we will specifically focused on heart-derived dECM.

### Properties of Decellularized Cardiac Tissue

Application of decellularization technique in cardiac tissue engineering has rapidly progressed in the past 10 years. Ott et al. (2008) reported for the first time the development of decellularized rat whole heart perfusing different solutions through the coronary access. The so-treated decellularized heart preserved the complex ECM composition as well as deprived of genetic material (Ott et al., 2008). Following this pioneering research, several studies have reported that, after decellularization, cardiac dECM retains intact geometry and vascular network of native heart, which makes it a suitable physiological platform for producing engineered construct for cardiac repair (Song and Ott, 2011; Tapias and Ott, 2014; Taylor et al., 2014; Wang et al., 2014). For all tissues and organs of the body, and especially for those of musculoskeletal compartment, ECM structure, ultrastructure and composition are mandatory aspects to be considered before a therapeutic application. In the recent years, mechanical properties of biomaterial scaffolds have been recognized as important player in influencing tissue repair, especially in organs such as cardiac and skeletal muscles (Singelyn and Christman, 2011; Hasan et al., 2014; Piccoli et al., 2016). The preservation of structural and mechanical characteristics of dECM after *in vivo* implantation could allow improvement in the mechanisms of cardiac repair and regeneration. From one hand, dECM could provide the infarcted myocardium with incisive mechanical compensation. In fact, tissue mechanical properties are mainly determined by ECM. From the other hand, the structural, ultrastructural and mechanical characteristics of dECM could serve as physical messenger for the delivered cells or infiltrated host cells to augment cardiovascular differentiation and tissue regeneration. Together with the mechanical properties, preservation of biochemical cues within dECM could be desirable for cell attachment, proliferation and stem cell differentiation, both *in vivo* and *in vitro*, as demonstrated in many studies (Singelyn and Christman, 2010; Singelyn et al., 2012; Wang and Christman, 2016). Proteomic approaches evidenced the retention of ECM proteins after decellularization using human myocardium tissue (de Castro Bras et al., 2013; Johnson et al., 2016). Among all the major components, such as collagens, laminin, elastin and glycosaminoglycans, several studies have reported that cardiac dECM also holds the soluble growth factors after decellularization process (Methe et al., 2014; Ferng et al., 2017). Some of these peptides and cytokines within the decellularized myocardium tissue are involved in cardiac homeostasis and remodeling, angiogenesis, survival, proliferation, differentiation and cell recruitment in response to inflammation (Di Meglio et al., 2017). In particular, neonatal murine cardiac dECM obtained from 1-day-old pups has been shown to trigger *in vitro* turnover in unresponsive cardiomyocytes, as enriched with Agrin and Tgf - b1 which have been described acting like ECM-associated mitogens by inhibiting the Hippo effector Yap, via the dystrophin glycoprotein complex (Bassat et al., 2017; Eroglu and Chien, 2017). Differently from the mechanical cues, the component types and their amounts in the dECM are applicable for dECM patches as well as injectable dECM hydrogels.

### Cardiac Extracellular Matrix-Derived Scaffolds and Hydrogels to Treat Cardiovascular Disease

Decellularized ECM-derived formulations, as alone or combined with cells/factors, have been described supporting cardiac repair in preclinical settings (Zisch et al., 2003; Seif-Naraghi et al., 2010; Sarig et al., 2016). One of the most remarkable obstacles in the classical cell-therapy vision is represented by the harsh cardiac environment following MI, which affects engraftment of transplanted cells and their capacity to *de novo* contribute to tissue repair. The fate of transplanted cells, in fact, is strongly affected by ischemic myocardium remodeling, including altered ECM anisotropy (Gupta et al., 1994; Carey et al., 2001). A promising therapeutic approach is represented by the inhibition of adverse post ischemic dilation to create a more suitable milieu that allows engrafted (or resident regenerating) cells to be functionally activated; myocardial or pericardial constructs are engineered to help preventing progression into heart failure and sustain cardiac function after acute myocardial impairment (Kameli et al., 2018; Zhang J. et al., 2018; Streeter and Davis, 2019).

Numerous experimental analyses confirmed that cardiac dECM may be considered an appealing source for the development of new myocardial repair strategies. First of all, thanks to the preservation of biomechanical properties, cardiac dECM patches could cope with the contraction/relaxation cycle of the heart, thus mechanically securing the infarcted area and counteracting fibrosis along with pathological remodeling (Carotenuto et al., 2019). Moreover, the important physical signals retained by the patches within their stiffness and texture are delivered to the resident cells while also sustaining diffusion of nutrients, and removal of pro-necrotic factors. It is important to stress, in fact, that stiffness and elastic modulus of pathological hearts are different from the healthy tissue (Arani et al., 2017), and that this aspect heavily influences cellular behaviors. In addition, since dECM is biocompatible and degradable, serves as a temporary scaffold that enables cell engraftment directly into necrotic and infarcted regions, overcoming the benefits of synthetic materials commonly used in the clinic. Indeed, evidences have been reported on cardiac dECM patch being able to support host tissue-driven reconstruction of a full-thickness right ventricular outflow tract defect in a rat model, after 16 weeks of treatment (Wainwright et al., 2012).

Given that dECM patches and scaffolds are delivered epicardially by exposed−chest surgery, better methods to enhance the therapeutic efficacy and reduce invasiveness are desirable. In this scenario, injectable cardiac-based biomaterials may be very appealing as exploitable by minimally invasive approach, with *in vivo* similar efficacy in respect to cardiac dECM patches (Seif-Naraghi et al., 2010; Toeg et al., 2013). For example, decellularized pericardium produced as a milled matrix, was injected into the cardiac left ventricle of healthy rats, demonstrating to form a fibrous, porous scaffold *in vivo* (Seif-Naraghi et al., 2010). When injected as stand-alone formulations in small animal model of MI, this biomaterial contributed to preserve cardiac function. Interestingly, on the basis of neonatal rodent increased ability to regenerate after myocardial injury, a recent research work showed that neonatal rodent cardiac dECM counteracted ventricular remodeling in adult mice following MI, underlining once again the importance of tissue specificity and the strong relationship between regeneration and cardiac tissue age also when considering the ECM (Wang et al., 2019). While most of the mechanisms behind the effectiveness of these injectable dECM-derived biomaterials *in vivo* have yet to be comprehensively examined, it is clear that porous scaffolds facilitate cell homing and neovascularization in ischemic regions. Seif-Naraghi et al. (2011) demonstrated that the variability within human cardiac tissue samples obtained after protein composition, glycosaminoglycan content, *in vitro* degradation, *in vivo* gelation, and microstructure analyses of 7 different human specimens, does not prevent them from being processed into injectable scaffold. Moreover, all these samples displayed similar fibrous and porous texture and cell infiltration after *in vivo* injection, prompting a possible application to limit detrimental remodeling after MI (Seif-Naraghi et al., 2011).

### Engineered Cardiac Extracellular Matrix-Derived Devices

dECM formulations as delivery platform for cardiovascular repair and regeneration are attracting growing attention. One of the most promising approach is the combination *in vitro* of natural scaffolds with patient-derived progenitor cells, in order to re-create tissue substitute before *in vivo* application. Healthy and responsive cells, in fact, are mandatory players in the regeneration game, especially when big portions of tissue are damaged. Their presence in the engineered scaffold before implantation results in tenability of the construct. In addition, cells increased their active bound and primed the dECM construct, according to their needs and behaviors (Chi Ting Au-Yeung et al., 2017). These interactions ensure an increased resistance of the engrafted construct and give also a good stabilization to the injured native tissue, rising the beneficial effect of this tissue engineering approach. Enhanced effects could be obtain with a multiple strategy, involving the injection of tissue specific cells, following the classical cell therapy approach, and a stromal cell-loaded patch, in which the engineered construct provides a microenvironment that stimulates vascular regeneration through prolonged secretion of paracrine factors and simultaneously ameliorates the engraftment of cells delivered locally and contributing to the rescue of cardiac function (Park et al., 2019).

At the same time, the use of natural derived hydrogels for the therapeutic administration of cells and factors has shown great efficacy and allowed a less invasive approach. On top of using bioactive dECM hydrogels as vehicle for the administration of putative cardiomyogenic precursors to optimize their retention and engraftment within the myocardial tissue (Bejleri et al., 2018; Bai et al., 2019; Kim et al., 2019), dECM scaffolds are becoming increasingly attractive as *in situ* releasing reservoir for secreted paracrine factors. In this scenario, loading of growth factors into biomaterial scaffolds increased their stability and activity. Data from a preclinical work indicate that delivery of a specific fragment of HGF in an ECM hydrogel supported neovascularization and limited ventricle dilation following MI (Zisch et al., 2003). The prolonged presence and the enhanced stability of the exogenous factor entrapped in the matrix material probably support and foster the native tissue response. In addition, the ECM hydrogel may also represent a working system to deliver cytokines to target cells similarly to endogenous factors physiologically requisitioned in the native cardiac ECM. This approach of secreted factors reservoir could be also obtained starting from *in vitro* cell culture material: human adipose dECM hydrogels as controlled-delivery system of factors secreted by adipose-derived stromal cells has been recently tested *in vitro* to demonstrate that different cell-derived paracrine factors could be delivered concurrently through a controlled-release approach on target cells in an *in vitro* wound healing model (van Dongen et al., 2019).

Likewise, dECM could be also envisaged as bioink for the precise and custom-made 3D-printing of bioactive scaffolds to deliver cells and/or their paracrine factors to the injured myocardium; indeed, 3D-printed bio-patches composed of cardiac ECM, human CPC, and gelatin methacrylate have been shown to be retained on rat hearts and mediated vascularization within 14 days from delivery. Notably, conditioned media from the bioengineered patches provided angiogenic effects, as detected by increased endothelial cell tube formation (Bejleri et al., 2018). Hence, by fostering cardio-active stem/precursor secretome potential via enhanced dECM delivery, we could define *ad hoc* paracrine cardiac therapy as future strategy (Figure 1). Furthermore, since dECM still maintains specific bioactive paracrine content *per se*, their EV profile is also gaining attention. Indeed, significant amount of EVs has been described to be released by cardiac ECM, namely ECM-EVs. Notably, ECM-EVs showed to be enriched with the cardiomyogenic miR-199a-3p, promoting cell division in isolated neonatal cardiomyocytes, as well as supporting electrical activity (An et al., 2017).

**FIGURE 2:**
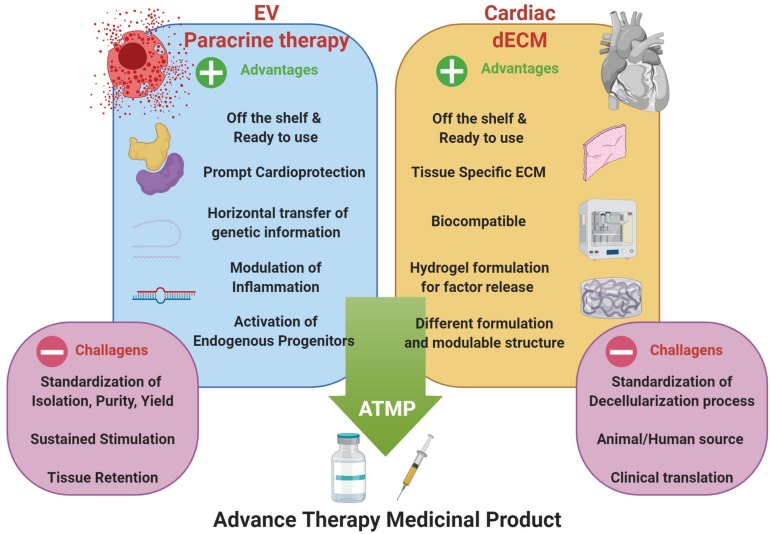
Optimization of EV-based paracrine therapy with dECM technology. Cardiac regenerative strategy based on the synergistic combination of EV-based beneficial effects and cardiac dECM formulations for putative future paracrine therapy, as ready-to-use and off-shelf advanced therapy medicinal product (ATMP); indeed, such innovative approach may overcome some of the major limits of EV-based strategy, such as tissue retention, tissue specific tropism and sustained, controlled release. Schematic was drawn using BioRender (https://app.biorender.com).

Therefore, the combination of cell-based strategies, such as paracrine therapy via EV administration, with the functionalization of dECM formulations, may represent an innovative approach in cardiac regenerative medicine for the next future. The analysis of biopolymer/hydrogel formulations to deliver therapeutically relevant EVs is gaining increasing attention. Some critical aspects limiting the clinical translation of therapeutic use of EVs could be improved through the synergistic combination with hydrogel-based or injectable cardiac dECM formulations; these challenges include the need for sustained and controlled release of EVs within the cardiac tissue over time and their local delivery via compatible cardiac-specific formulations. To date, both xenogeneic and allogeneic dECM have been used in pre-clinical and clinical research with different results, but availability of donor tissues is often limited. From one hand, xenogeneic heart from big animals (e.g., porcine or bovine tissues) are easy to obtain, but may carry residual immunogenicity and may be contaminated with biological agents. On the other hand, human allogeneic tissues would be the ideal material to obtain dECM suitable for implantation or generation of hydrogel. Together with cadaver organ donation, human tissue Biobanks are important sources to augment organ availability (Porzionato et al., 2018), especially because the different formulations of cardiac dECM do not require all the stringent regulations necessary for the classic organ donation for transplant purposes. Moreover, general standard decellularization protocols should be set up before clinical application. Hence, in the next future, by exploiting EV biology with cardiac dECM technology we could envisage a possible novel ATMP (Figure 2). Currently, only a couple proof-of-principle studies have been performed on the functional encapsulation of EVs within dECM-derived hydrogels; porcine-derived dECM hydrogels have been tested as platform for sustained delivery of microRNAs and CPC-EVs to validate controlled release along with EV anti-apoptotic bioactivity *in vitro* (Hernandez et al., 2018). More recently, the feasibility of EV transport in hydrogels and a decellularized matrix has been demonstrated. Engineered biomaterials with specific matrix mechanical properties, as stress relaxation, showed to influence EV retention, while higher crosslinking density support their diffusion (Lenzini et al., 2020).

### Future Perspectives: Translation Into the Clinical Scenario

When considering possible clinical translation of the most recent advances in cardiovascular research, there are relevant open questions that still need to be addressed. These include the optimal time point of intervention following injury/during disease, and the most suitable strategy to pursue in terms of enhancement of cardiac repair over stimulation of endogenous regeneration, according to the cardiovascular patient’s specific needs (i.e., acute MI or ischemic coronary disease versus chronic cardiomyopathy). In this scenario, innovative cardiac regenerative strategies, based on either paracrine or cell-therapy approaches, may be challenged by poor cardiac tropism of cells/factors injected systemically and may require local or intra-myocardial administration to enhance their effects. Novel bio-scaffold/hydrogel formulations can increase and enhance retention and release; yet they may depend on topical delivery, and thus being indicated as concomitant strategy during heart surgery or percutaneous coronary intervention/angioplasty. While not ideal for any kind of cardiovascular patient, they can still be therapeutically relevant for acute treatments, such as prompt intervention on patients experiencing MI. The functional combination of prompt EV modulatory effects with the tunable biological properties of dECM hydrogels may represent an ideal delivery platform to enhance intrinsic mechanisms of cardiac restoration. Another relevant cardiovascular scenario is then represented by congenital heart defects, which may affect up to ca. 35.000 newborns annually, significantly impacting on patient morbidity and mortality (Rossano et al., 2012). Palliative surgery with use of cardiac prosthetic devices is the elective options in these patients, although it does not offer a permanent solution, as follow-up interventions are required and may not be resolutive, with children ultimately developing heart failure. Moreover, constructs or supports have been often dispensed to the outer cardiac surface during open−chest surgery. Future efforts should provide less interfering systems based on implementing biomaterials as topical reservoir of bioactive factors, such as self-assembling tissue-specific hydrogels or other tunable and smart biomaterials. In this perspective, the use of cardiac dECM bio-printed platforms/bio-scaffolds to deliver cells/paracrine factors at the time of reconstructive surgery, could represent a synergic and implementing strategy, to provide endogenous repair and regeneration of the young cardiac tissue.

## Author Contributions

EM: manuscript writing, analytical discussion of decellularized extracellular matrix biology and applications, and figure design. PG: manuscript writing, critical discussion of stem cell paracrine biology. RQ: manuscript writing, contribution to critical discussion on cardiac repair. MP and SB: conception and design, manuscript writing and supervision. All authors reviewed the manuscript and approved it.

## Conflict of Interest

The authors declare that the research was conducted in the absence of any commercial or financial relationships that could be construed as a potential conflict of interest.
